# Association of Childhood Lead Exposure With Adult Personality Traits and Lifelong Mental Health

**DOI:** 10.1001/jamapsychiatry.2018.4192

**Published:** 2019-01-23

**Authors:** Aaron Reuben, Jonathan D. Schaefer, Terrie E. Moffitt, Jonathan Broadbent, Honalee Harrington, Renate M. Houts, Sandhya Ramrakha, Richie Poulton, Avshalom Caspi

**Affiliations:** 1 Department of Psychology and Neuroscience, Duke University, Durham, North Carolina; 2 Center for Genomic and Computational Biology, Duke University, Durham, North Carolina; 3 Department of Psychiatry and Behavioral Sciences, Duke University, Durham, North Carolina; 4 King’s College London, Social, Genetic, and Developmental Psychiatry Centre, Institute of Psychiatry, Psychology, & Neuroscience, London, United Kingdom; 5 Sir John Walsh Research Institute, Faculty of Dentistry, University of Otago, Dunedin, New Zealand; 6 Dunedin Multidisciplinary Health and Development Research Unit, Department of Psychology, University of Otago, Dunedin, New Zealand

## Abstract

**Importance:**

Millions of adults now entering middle age were exposed to high levels of lead, a developmental neurotoxin, as children. Although childhood lead exposure has been linked to disrupted behavioral development, the long-term consequences for adult mental and behavioral health have not been fully characterized.

**Objective:**

To examine whether childhood lead exposure is associated with greater psychopathology across the life course and difficult adult personality traits.

**Design, Setting, and Participants:**

This prospective cohort study was based on a population-representative birth cohort of individuals born between April 1, 1972, and March 31, 1973, in Dunedin, New Zealand, the Dunedin Multidisciplinary Health and Development Study. Members were followed up in December 2012 when they were 38 years of age. Data analysis was performed from March 14, 2018, to October 24, 2018.

**Exposures:**

Childhood lead exposure ascertained as blood lead levels measured at 11 years of age. Blood lead levels were unrelated to family socioeconomic status.

**Main Outcomes and Measures:**

Primary outcomes were adult mental health disorder symptoms assessed through clinical interview at 18, 21, 26, 32, and 38 years of age and transformed through confirmatory factor analysis into continuous measures of general psychopathology and internalizing, externalizing, and thought disorder symptoms (all standardized to a mean [SD] of 100 [15]) and adult personality assessed through informant report using the Big Five Personality Inventory (assessing neuroticism, extraversion, openness to experience, agreeableness, and conscientiousness) at 26, 32, and 38 years of age (all scores standardized to a mean [SD] of 0 [1]). Hypotheses were formulated after data collection; an analysis plan was posted in advance.

**Results:**

Of 1037 original study members, 579 (55.8%) were tested for lead exposure at 11 years of age (311 [53.7%] male). The mean (SD) blood lead level was 11.08 (4.96) μg/dL. After adjusting for study covariates, each 5-μg/dL increase in childhood blood lead level was associated with a 1.34-point increase (95% CI, 0.11-2.57; *P* = .03) in general psychopathology, driven by internalizing (b = 1.41; 95% CI, 0.19-2.62; *P* = .02) and thought disorder (b = 1.30; 95% CI, 0.06-2.54; *P* = .04) symptoms. Each 5-μg/dL increase in childhood blood lead level was also associated with a 0.10-SD increase in neuroticism (95% CI, 0.02-0.08; *P* = .02), a 0.09-SD decrease in agreeableness (95% CI, −0.18 to −0.01; *P* = .03), and a 0.14-SD decrease in conscientiousness (95% CI, −0.25 to −0.03; *P* = .01). There were no statistically significant associations with informant-rated extraversion (b = −0.09; 95% CI, −0.17 to 0.004; *P* = .06) and openness to experience (b = −0.07; 95% CI, −0.17 to 0.03; *P* = .15).

**Conclusions and Relevance:**

In this multidecade, longitudinal study of lead-exposed children, higher childhood blood lead level was associated with greater psychopathology across the life course and difficult adult personality traits. Childhood lead exposure may have long-term consequences for adult mental health and personality.

## Key Points

**Question:**

Is childhood lead exposure associated with the risk of mental illness or difficult personality traits in adulthood?

**Findings:**

In this longitudinal cohort study of 579 New Zealand children followed up for more than 30 years, greater lead exposure in childhood was significantly associated with greater psychopathology across the life course and with difficult personality traits in adulthood.

**Meaning:**

Childhood lead exposure may have long-term consequences for adult mental health and personality.

## Teaser

This cohort study assesses whether exposure to lead during childhood is associated with lifelong mental health disorder symptoms and difficult personality traits in adulthood.

## Introduction

Millions of adults now entering middle age were exposed to high levels of lead as children,^1^ a phenomenon that accompanied the peak use of lead in gasoline worldwide from the 1940s through the early 1990s.^2^ From 1976 to 1980, the average child living in the United States had blood lead levels (BLLs) 3 times higher (>15 μg/dL)^1^ than the current reference value for clinical attention (5 μg/dL).^3^ Lead-exposed children experience disrupted cognitive and behavioral development,^4^ with childhood lead exposure linked to lower child IQ,^5^ poorer academic achievement,^6^ and greater rates of child behavior problems, particularly inattention, hyperactivity, and antisocial behavior.^7,8,9^ Meanwhile, adults exposed to lead are at increased risk of developing some psychiatric conditions.^10,11,12,13^ Although follow-up studies^14,15^ of lead-tested children have reported the persistence of lead-related cognitive deficits well into adulthood, apart from antisocial outcomes, the long-term mental and behavioral health consequences of early-life lead exposure have not been fully characterized.

To our knowledge, 2 studies^16,17^ have undertaken long-term follow-up in lead-exposed children to determine whether early behavior problems persist or evolve into adult mental health concerns (a larger number of studies^18,19,20,21,22^ have examined whether adolescents and young adults exposed to lead as children display more antisocial and criminal behaviors, with most studies, although not all, suggesting that they do). A US study^16^ that used linked health records and clinical interviews to identify cases of psychosis in 2 lead-tested child cohorts born in the late 1960s (N = 200; age range, 30-35 years at follow-up) reported a 2-fold increased risk of schizophrenia spectrum disorder in adulthood for individuals with high BLLs as children (approximately >15 μg/dL). Another study, which is to our knowledge the only comprehensive adult psychiatric follow-up study^17^ conducted in a lead-tested child cohort (N = 210; cohort born in the early 1980s), reported greater social phobia, anxiety, and substance abuse problems in adulthood (mean age at follow-up, 26.3 years) for Australian women who had greater BLLs as children, although all associations were attenuated to nonsignificance by statistical adjustment for study covariates, including parental educational and occupational attainment.

This existing evidence base has limitations. First, because of small sample sizes, these studies had limited power to detect effects. Second, because they considered only specific disorders (eg, schizophrenia) or relied on right-hand censored, single–time point clinical interviews to assess psychiatric problems, these studies likely underdetected episodes of illness and overlooked disorders that have a pattern of reoccurrence.^23^ Third, it is now appreciated that most psychiatric disorders are dimensional constructs, not discrete categorical entities.^24^ Individuals who meet criteria for one disorder typically also meet criteria for others both cross-sectionally and across the life course.^25,26,27^ Empirical evidence suggests that psychiatric illnesses can be represented by 3 higher-order dimensions—internalizing, externalizing, and thought disorders (eg, psychotic experiences)^28^—that are intercorrelated and may reflect a common liability toward psychopathology in general, labeled the *p factor*.^29,30,31^ The p factor may be a particularly appropriate outcome for studies that link environmental toxins to mental disorder because (1) the few previous studies^10,11,13,16,17^ of lead and psychopathology suggest that lead exposure may increase the risk of internalizing, externalizing, and thought disorders, without particular specificity, and (2) the continuous and omnibus nature of the p factor allows investigators to easily test for dose-effect relationships.

Although epidemiologists have hypothesized a relationship between child lead exposure and adult psychopathology, another way of considering the link between lead and behavioral dysfunction focuses on personality features that may impair an individual's capacity to lead a happy, successful life. Decades of research using the Big Five framework to represent the 5 broadest factors of personality^32^ have identified a combination of traits, including poor impulse control, high antagonism, and a tendency toward negative emotionality, that have a detrimental effect on love, work, and health and that appear to predispose individuals to psychiatric illness.^33,34,35^ Few studies have examined personality traits in association with lead exposure, but adults occupationally exposed to lead have reported feeling angrier and more tired, tense, and depressed than their less exposed peers^36,37^; these emotional symptoms seem to improve with the abatement of lead exposure.^38^ In the one cohort of lead-tested children who received personality testing (born in Cincinnati, Ohio, in the early 1980s; aged 19-24 years at follow-up), young adults with greater childhood lead exposure tested higher, on average, than cohort peers on a self-report inventory of psychopathic traits, such as impulsivity and egocentricity.^39^ Alterations in emotion regulation and adult personality have consequently been proposed as explanatory mechanisms for the reported link between childhood BLLs and adolescent delinquency^40^ and young adult criminal arrests^41^ also observed in this Cincinnati cohort.

**Figure 1.**
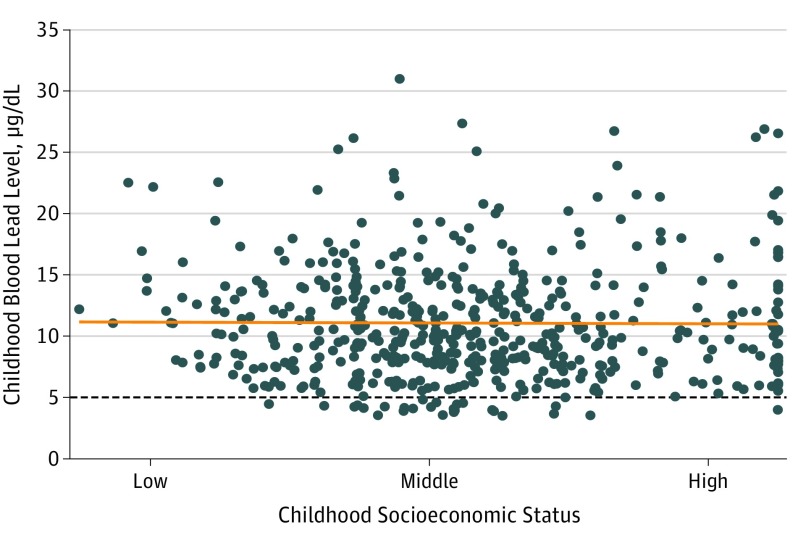
Association of Childhood Blood Lead Level at 11 Years of Age With Child Family Socioeconomic Status There was no significant social gradient in lead exposure in the Dunedin study children (*r* = −0.01; 95% CI, −0.08 to 0.07; *P* = .86); high blood lead levels were observed among children from all socioeconomic status groups. Childhood socioeconomic status was determined through the 6-point Elley-Irving scale (categories 1 and 2 [low status], 3 and 4 [middle status], and 5 and 6 [high status]), which codes the occupations and associated income and educational levels of members’ parents. The orange line depicts the nonsignificant association between child blood lead levels and childhood socioeconomic status. The dotted line depicts the current Centers for Disease Control and Prevention child blood lead level reference value for clinical attention (5 μg/dL).^3^ A total of 554 (94.0%) tested study members had blood lead levels above the current reference value.

With this study, we undertook, to our knowledge, the longest and largest psychiatric follow-up to date in a cohort of adults who were lead exposed and lead tested as children, as well as the only follow-up to use (1) repeated clinical interviews assessing psychopathology symptoms across adulthood up to 38 years of age; (2) comprehensive, dimensional measures of psychopathology that account for severity, comorbidity, and reoccurrence; and (3) a broad measure of adult personality (Big Five Personality Inventory)^32^ that did not rely on self-report. We conducted these follow-ups in a sample in which the extent of children's exposure to lead was unrelated to their socioeconomic origins^15^ (Figure 1), removing a potentially important confounder that is present in most studies of children and lead.^42^

## Methods

### Study Design and Population

Members were members of the Dunedin Multidisciplinary Health and Development Study, a longitudinal investigation of health and behavior in a birth cohort. The full cohort comprised all individuals born between April 1, 1972, and March 31, 1973, in Dunedin, New Zealand, who were eligible based on residence in the province and who participated in the first assessment at 3 years of age. The cohort represented the full range of socioeconomic status in the general population of New Zealand's South Island.^43^ With regard to adult health, the cohort matched the New Zealand National Health and Nutrition Survey on key indicators (eg, body mass index, smoking, and visits to a physician).^43^ The cohort was primarily white; less than 7% self-identified as having nonwhite ancestry, matching the demographics of the South Island.^43^ Assessments were performed at birth and 3, 5, 7, 9, 11, 13, 15, 18, 21, 26, and 32 years of age, and the most recent data collection was completed in December 2012, when members were 38 years of age. Data analysis was performed from March 14, 2018, to October 24, 2018. Written informed consent was obtained from parents and cohort members, and data were deidentified. Study protocols were approved by the Southern Health and Disability Ethics Committee at the New Zealand Ministry of Health and The Duke University Health System Institutional Review Board for Clinical Investigations.

### Measures

#### Childhood BLLs

Approximately 30-mL venous blood samples were obtained at 11 years of age from 579 of the 803 children (72.1%) who participated in the assessment performed at the Dunedin Multidisciplinary Health and Development Research Unit and who freely agreed to provide a blood sample. An additional 122 children were assessed in their schools, where blood samples could not be obtained. Whole-blood samples were analyzed through graphite fumance atomic absorption spectrophotometry. Details on the method of blood collection, storage, and analysis have been described previously.^9^

#### Assessment of Symptoms of Mental Disorder

The Dunedin study longitudinally ascertains mental disorders every 2 to 6 years, interviewing members about past-year symptoms. We also used life-history calendar interviews to ascertain indicators of mental disorder that occur in the gaps between assessments, including inpatient treatment, outpatient treatment, or spells taking prescribed psychiatric medication (indicators that are salient and recalled more reliably than individual symptoms). Life-history calendar data indicate that virtually all members with a disorder consequential enough to be associated with treatment have been detected in our net of past-year diagnoses made at 18, 21, 26, 32, and 38 years. Specifically, we identified only 11 people who reported treatment but had not been captured in our net of diagnoses from 18 to 38 years of age (most of whom experienced brief postnatal depression).

Psychopathology symptoms were assessed through private structured interviews using the *Diagnostic Interview Schedule*^44^ at 18, 21, 26, 32, and 38 years of age. Interviewers were health care professionals, had completed a 2-week training course to criterion, and were retrained periodically as needed throughout data collection. We studied *Diagnostic and Statistical Manual of Mental Disorders* (*DSM*)–defined symptoms of the following disorders that were repeatedly assessed in our longitudinal study: alcohol dependence, cannabis dependence, dependence on hard drugs, tobacco dependence (assessed with the Fagerström Test for Nicotine Dependence),^45^ conduct disorder, major depression, generalized anxiety disorder, fears and/or phobias, obsessive compulsive disorder, mania, and positive and negative schizophrenia symptoms. Ordinal measures represented the number of the 7 (eg, mania, generalized anxiety disorder) to 10 (eg, alcohol dependence, cannabis dependence) possible *DSM*-defined symptoms associated with each disorder. Fears and/or phobias were assessed as the count of diagnoses for simple phobia, social phobia, agoraphobia, and panic disorder that a study member reported at each assessment. Symptoms were assessed without regard for hierarchical exclusionary rules to facilitate the examination of comorbidity. Each of the 11 disorders were assessed at least 3 times. The past-year prevalence rates of psychiatric disorders in the Dunedin cohort are similar to prevalence rates in nationwide surveys of the United States and New Zealand.^23,46^

#### Structure of Psychopathology From 18 to 38 Years of Age

The methods used to compute the hierarchical measures of psychopathology in the Dunedin cohort have been described previously.^29^ In brief, we used confirmatory factor analysis to calculate factor scores that represent internalizing (with loadings from depression, anxiety, and fear and/or phobia symptoms), externalizing (with loadings from substance dependence and conduct disorder symptoms), and thought disorders (with loading from obsessive-compulsive, manic, and psychotic symptoms), as well as general psychopathology (ie, the p factor; with loadings from all 11 assessed disorders). Fit indexes met criteria for good model fit. For expository purposes, we scaled study members’ scores on all factors to a mean (SD) of 100 (15). These measures are further described in the eMethods and eFigure in the Supplement.

#### Adult Personality

At 26, 32, and 38 years of age, study members nominated people who knew them well. These informants were mailed questionnaires and asked to describe each study member using a 25-item version of the Big Five Personality Inventory, which measured the personality traits of neuroticism, extraversion, openness to experience, agreeableness, and conscientiousness.^47^ We created cross-age composites for each of the traits. In the analysis sample, these measures correlated with study members’ scores for general psychopathology (*r* = 0.38, *P* < .001 for neuroticism; *r* = 0.07, *P* = .10 for extraversion; *r* = 0.07, *P* = .12 for openness to experience; *r* = −0.27, *P* < .001 for agreeableness; and *r* = −0.29, *P* < .001 for conscientiousness).

#### Child Externalizing and Internalizing Problems

At 11 years of age, parents and teachers completed the Rutter Child Scale,^48^ a questionnaire that inquires about the major areas of behavioral and emotional functioning during the past year. Parents and teachers rated each behavior on the Rutter Child Scale as “does not apply” (score of 0), “applies somewhat” (score of 1), or “certainly applies” (score of 2). Child externalizing problems were assessed using scores for the 8-item antisocial scale and scores for 4 items that address hyperactivity. Items on the antisocial scale describe children who frequently fight, bully other children, lie, disobey, steal, destroy belongings, and have irritable tempers. Items that contribute to the measurement of hyperactivity describe children who are “very restless,” “hardly ever still,” “squirmy,” “fidgety,” and unable to “settle into anything.” Child internalizing problems were assessed using scores on 6 items that describe children who “worry about many things” and “often appear miserable,” “unhappy,” and “tearful.” Details about the reliability and validity of the parent and teacher versions of the scale have been described previously.^49,50^ Parent and teacher scores were averaged.

#### Covariates

Study covariates included family-level risk factors known to relate to childhood lead exposure or adult psychopathology and personality, including family socioeconomic status, maternal IQ, and family history of mental illness. These measures are described in the eMethods and eFigure in the Supplement.

### Comparison of Members Who Were Tested for Lead Exposure at 11 Years of Age vs Those Not Tested

A total of 579 study members had been tested for lead exposure during childhood (55.8% of the full cohort). Study members with and without (n = 458 [44.2%]) BLL data were similar on all study covariates, including their social class origins, their mother's IQ scores, and their family history of mental illness. However, as a group, those without BLL data had greater internalizing problems at 11 years of age (mean of −0.06 *z*-scored units for children with BLL data; mean of 0.10 *z*-scored units for children without BLL data; difference of 0.16; *P* = .02).

### Statistical Analysis

First, we tested the association between childhood BLLs and adult general psychopathology using ordinary least-squares multiple linear regression. We also tested for specificity in the association between childhood lead exposure and adult psychopathology by examining whether BLLs were associated with scores on the internalizing, externalizing, and thought disorder factors. Each outcome was examined using 2 models: (1) a sex-adjusted model in which the outcome was regressed on childhood BLL and sex and (2) a fully adjusted model that included all covariates. We used these same models to test associations between childhood BLLs and scores on informant-reported measures of adult personality. Post hoc sensitivity tests were also conducted to examine possible sex differences in the association between childhood BLLs and the adult outcome measures.^51^ The models specified above were rerun with a sex × lead interaction term included; these terms were nonsignificant in all models (*P* values ranged from .13 to .66).

Second, we repeated these analyses using measures of childhood externalizing and internalizing problems (ie, antisocial behavior, hyperactivity, and internalizing problems) in place of adult outcomes. These models allowed us to test whether the association between lead exposure and psychopathology could be seen as early as 11 years of age.

Only members who had complete data on all covariates for each outcome were included in each model; no data were imputed. For adult psychopathology, 551 members (95.2%) were analyzed; for adult personality, 539 members (93.1%) were analyzed; and for childhood externalizing and internalizing problems, 552 members (95.3%) were analyzed. Lead level was analyzed as a continuous measure and is presented here in 5-μg/dL units with 95% CIs, which correspond to approximately 1 SD of BLL in the cohort. Association of lead exposure in childhood with adult personality differences is presented with regression coefficients and 97% CIs. *P* values were generated from *t* tests for the null hypothesis. A 2-tailed *P* < .05 was considered to be statistically significant.

## Results

### Association of Lead Exposure in Childhood With Psychopathologic Measures In Adulthood

**Figure 2.**
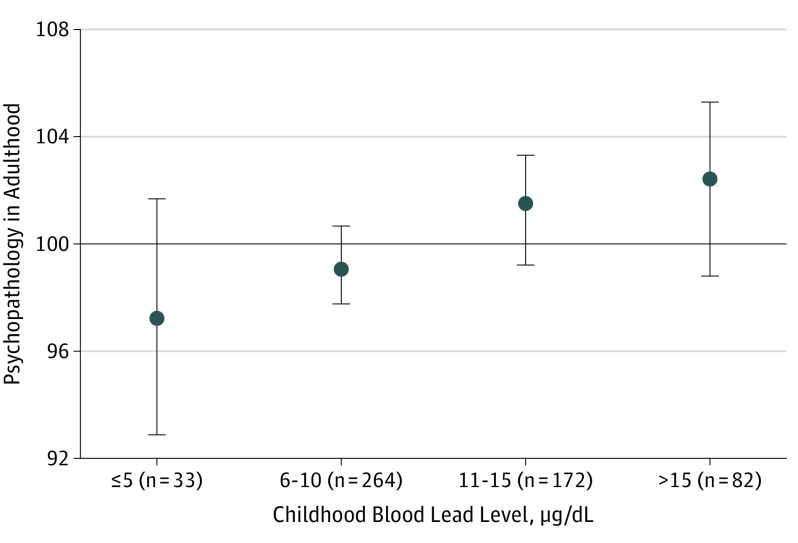
Association of Childhood Blood Lead Level at 11 Years of Age With Adult General Psychopathology at 38 Years of Age (Unadjusted for Covariates) The mean general psychopathology scores (circles) in adulthood with 95% CIs (error bars) by childhood blood lead level are shown. Each 5-μg/dL–higher level of blood lead in childhood was associated with an additional 1.49-point higher score (95% CI, 0.22-2.77; *P* = .02) in adult general psychopathology on a scale standardized to a mean (SD) of 100 (15) (horizontal line). Of the 579 study members with childhood blood lead level measured, 551 (95.2%) also had present data on all the covariates and the psychopathology outcome measures.

Of 1037 original study members, 579 (55.8%) were tested for lead exposure at 11 years of age (311 [53.7%] male). Child BLLs ranged from 4 to 50 μg/dL (mean [SD], 11.08 [4.96] μg/dL; to convert to micromoles per liter, multiply by 0.0483). A total of 544 study members (94.0%) had BLLs above the current reference value for clinical attention (5 μg/dL).^3^ Figure 2 depicts the mean adult general psychopathology scores of members at each childhood BLL. Members with childhood BLLs above the historical international level of concern for clinical attention (>10 μg/dL)^3^ tested a mean of 2.52 points higher (95% CI, 0.14-4.90; *P* = .04) on general psychopathology than their peers with lower BLLs (after adjusting for covariates, 2.30 points higher; 95% CI, −0.02 to 4.62; *P* = .05).

Results from the multiple linear regressions testing associations between BLL at 11 years of age and psychopathology from 18 to 38 years of age are given in the Table. After adjusting for covariates, each 5-μg/dL increase in childhood BLL was associated with a 1.34-point increase (95% CI, 0.11-2.57; *P* = .03) in general psychopathology. Examination of models testing associations between BLLs and factor scores for internalizing, externalizing, and thought disorder symptoms indicated that the association between BLLs and general psychopathology was driven primarily by associations between childhood BLL and internalizing and thought disorder symptoms. After adjusting for covariates, each 5-μg/dL increase in childhood BLL was associated with a 1.41-point increase (95% CI, 0.19-2.62; *P* = .02) in internalizing and a 1.30-point increase (95% CI, 0.06-2.54; *P* = .04) in thought disorder symptoms.

**Table.** Association Between Childhood Blood Lead Level and Adult Psychopathology, Adult Personality Traits, and Childhood Externalizing and Internalizing Problems^a^

Variable	Sex Adjusted		Fully Adjusted	
	b (95% CI)	*P* Value	b (95% CI)	*P* Value
Adult psychopathology^b^				
General psychopathology	1.49 (0.22 to 2.77)	.02	1.34 (0.11 to 2.57)	.03
Externalizing symptoms	0.80 (−0.47 to 2.06)	.21	0.73 (−0.52 to 1.97)	.25
Internalizing symptoms	1.57 (0.30 to 2.83)	.02	1.41 (0.19 to 2.62)	.02
Thought disorder symptoms	1.44 (0.16 to 2.72)	.03	1.30 (0.06 to 2.54)	.04
Adult personality traits (Big Five Personality Inventory)^c^				
Neuroticism	0.10 (0.02 to 0.19)	.01	0.10 (0.02 to 0.18)	.02
Extraversion	−0.08 (−0.17 to 0.01)	.09	−0.09 (−0.17 to 0.004)	.06
Openness to experience	−0.07 (−0.16 to 0.03)	.17	−0.07 (−017 to 0.03)	.15
Agreeableness	−0.09 (−0.17 to −0.003)	.04	−0.09 (−0.18 to −0.01)	.03
Conscientiousness	−0.14 (−0.25 to −0.03)	.01	−0.14 (−0.25 to −0.03)	.01
Childhood externalizing and internalizing problems^d^				
Antisocial behavior	0.11 (0.03 to 0.19)	.01	0.10 (0.02 to 0.18)	.02
Hyperactivity	0.17 (0.08 to 026)	<.001	0.16 (0.07 to 0.25)	<.001
Internalizing problems	0.12 (0.03 to 0.20)	.01	0.11 (0.02 to 0.20)	.01

^a^ Covariates in the fully adjusted model were sex, childhood socioeconomic status, maternal IQ, and family history of mental illness. Of the 579 study members with childhood blood lead level measured, 551 (95.2%) had present data on all the covariates and the psychopathology outcome measures, 539 (93.1%) had present data on all the covariates and the personality outcome measures, and 552 (95.3%) had present data on all the covariates and the childhood emotion and behavior outcome measures. Regression coefficients indicate change in outcome per 5-μg/dL increase in childhood blood lead level.

^b^ General psychopathology and the constituent psychiatric spectra are standardized to a mean (SD) of 100 (15).

^c^ The Big Five Personality Inventory traits scores are standardized to a mean (SD) of 0 (1).

^d^ Childhood antisocial behavior, hyperactivity, and internalizing problem scores are standardized to a mean (SD) of 0 (1).

### Association of Lead Exposure in Childhood With Adult Personality Differences

Results from the multiple linear regressions testing associations between childhood BLL and the informant-reported measures of adult personality are given in the Table. Consistent with the adult psychopathology results, after adjustment for covariates, study members with higher BLLs at 11 years of age were viewed in adulthood by their informants as more neurotic (b = 0.10; 95% CI, 0.02-0.08; *P* = .02), less agreeable (b = −0.09; 95% CI, −0.18 to −0.01; *P* = .03), and less conscientious (b = −0.14; 95% CI, −0.25 to −0.03; *P* = .01). There were no statistically significant associations with informant-rated extraversion (b = −0.09; 95% CI, −0.17 to 0.004; *P* = .06) and openness to experience (b = −0.07; 95% CI, −0.17 to 0.03; *P* = .15).

### Early Detection of Lead-Related Psychiatric Differences

After the detection of significant associations between child BLLs and both adult psychopathology symptoms and difficult adult personality traits, we tested whether psychiatric problems related to lead exposure could be detected as early as 11 years of age, when BLLs were assessed. The Dunedin study reported in 1988 that children with higher BLLs at 11 years of age scored higher on concurrent parent-report measures of hyperactivity and inattention symptoms.^9^ We tested whether study members with higher BLLs at 11 years of age also scored higher on measures at 11 years of age that assessed a broader suite of early-life externalizing and internalizing problems, including parent- and teacher-report measures of antisocial behavior, hyperactivity, and internalizing problems. We found that they did score higher (Table), suggesting that the association between lead exposure and psychopathology may begin to manifest broadly well before adulthood.

## Discussion

This multidecade, longitudinal analysis of the association between childhood BLLs and adult mental health and personality generated 3 findings. First, across nearly 3 decades of follow-up, childhood BLLs were associated with higher levels of general psychopathology, driven primarily by greater rates of internalizing and thought disorder symptoms. Second, childhood BLLs were associated with higher neuroticism, lower agreeableness, and lower conscientiousness. Third, childhood BLLs were associated with greater externalizing and internalizing symptoms assessed contemporaneously with BLL measurement at 11 years of age. Each of these findings remained significant after adjusting for members’ social class backgrounds, their mothers’ IQs, and their family histories of mental illness.

These results suggest that early-life lead exposure in the era of leaded gasoline experienced by individuals who are currently adults may have contributed to subtle, lifelong differences in emotion and behavior that are detectable at least up to 38 years of age. Are these differences clinically or practically meaningful? On the one hand, the effect sizes reflecting the associations between childhood lead exposure and adult psychopathology and personality difficulties are small (approximately *r* = 0.08). This size is approximately one-third the size of the association seen in the Dunedin study between psychopathology and other modifiable (eg, childhood maltreatment, *r* = 0.21) and nonmodifiable (eg, family history of mental illness, *r* = 0.23) risk factors.^29^ Childhood lead exposure may not be a major etiologic factor in adult psychiatric disease today. On the other hand, compared with other findings from this sample, the associations reported herein are similar to those reported for lead and IQ^15^ and are stronger than those reported for lead and criminal offending.^20^ On a population basis, even modest alterations in risk can lead to significant shifts in the overall burden of disease.

The finding that associations between childhood BLLs and psychopathology symptoms were observable as early as the age of BLL testing suggests that lead-related alterations in emotion and behavior, however modest, likely emerge early and persist across the life course. Of note, in childhood, these psychopathology symptoms tended to involve more externalizing symptoms, particularly hyperactivity, whereas in adulthood, they tended to involve more internalizing symptoms. This finding suggests that lead-related alterations in emotion and behavior may demonstrate heterotypic continuity in their psychiatric presentation,^52^ with either one class of psychiatric disorders creating conditions that lead to another class (eg, when hyperactivity elicits harsh parenting, it may lead to anxiety and depression) or else the same underlying condition (eg, a general liability to psychopathology) presenting differently across different developmental windows.^29,53^

The association between childhood lead exposure and adult personality traits also suggests that lead-related differences in adult emotion and behavior can be detected not only by asking individuals to self-report on their mental health symptoms but also by simply asking informants who know them well to describe their behavior. Childhood lead exposure may alter how people behave toward or are perceived by others across their lives. In other studies,^33,35,54,55^ the blend of personality traits seen in adults exposed to lead as children has been associated with a number of poor life outcomes, including more psychopathology, worse physical health, less job satisfaction, and troubled interpersonal relationships.

The present study has implications for future research, public policy, and clinical practice. For researchers, these findings add further evidence to the suggestion that environmental toxins may affect important life outcomes through subtle changes in the way that individuals feel and behave. Future toxicologic studies should consider assessing these subjective outcomes alongside more objective ones, such as physical health. For policymakers and practitioners, the findings suggest that the generation of adult patients with a history of childhood lead exposure may benefit from increased screening and access to mental health services.^56^ As the generation of lead-exposed individuals age, it is also possible that bone loss during menopause and osteoporosis may result in childhood lead stored in bone being recirculated throughout the body, suggesting the testable hypothesis that the long-term consequences of childhood lead exposure may evolve or expand over time.^57^ It is possible that the pediatric challenges of the past may represent emerging concerns for geriatric psychiatry.

### Limitations

This study has limitations. First, it used a single, predominantly white cohort born in the 1970s; therefore, its results will require replication in other samples from other countries. Second, although child BLLs in this New Zealand cohort are similar to those recorded in other developed countries at the time of testing,^58,59^ the high historical levels of lead exposure experienced by the Dunedin study members may not generalize to the relatively lower levels of exposure that are more common for children in developed countries today. Nevertheless, children in many developed and developing countries still experience high lead exposure from contaminated water, soil, paints, and pipes.^60,61^ Third, there was only one time point of lead testing, which precluded evaluation of sensitive periods for associations of lead with behavior or of the effects of cumulative lead dose by adulthood. Fourth, this study was observational and does not establish a causal relationship between lead and the tested outcomes.

## Conclusions

In this multidecade, longitudinal study of lead-exposed children, higher childhood BLLs were associated with more psychopathology across the life course and difficult adult personality traits. Childhood lead exposure may have long-term psychiatric and behavioral consequences.

## Notes

Supplement. **eMethods.** Supplemental Material **eFigure.** Two Alternative Correlational Structures of Early-Adult Psychopathology
