# Supplementary material for: Association of Childhood Lead Exposure With Adult Personality Traits and Lifelong Mental Health
Source: JAMA Psychiatry. 2019 Jan 23;76(4):418–25. doi: 10.1001/jamapsychiatry.2018.4192 (PMC6450277; doi:10.1001/jamapsychiatry.2018.4192)
Supplement: Supplement. — eMethods. Supplemental Material eFigure. Two Alternative Correlational Structures of Early-Adult Psychopathology [file jamapsychiatry-76-418-s001.pdf]

Supplementary Online Content

Reuben A, Schaefer JD, Moffitt TE, et al. Association of childhood lead exposure with adult personality traits and lifelong mental health. *JAMA Psychiatry*. Published online January 23, 2019. doi:10.1001/jamapsychiatry.2018.4192

eMethods. Supplemental Material

eFigure. Two Alternative Correlational Structures of Adult Psychopathology

This supplementary material has been provided by the authors to give readers additional information about their work.

© 2019 Reuben A et al. *JAMA Psychiatry.*

eMethods. Supplemental Material

The structure of psychopathology

The methods used to compute our hierarchical measures of psychopathology in the Dunedin cohort have been described previously.[1] Briefly, we used confirmatory factor analysis to compute two standard models that are frequently used to examine hierarchically-structured constructs: a Correlated-Factors Model with 3 factors (representing Internalizing, Externalizing, and Thought Disorders; **eFigure A**), and a Bi-Factor Model specifying a General Psychopathology factor (labeled "p", **eFigure B**). These models included our 11 observed variables: alcohol dependence, cannabis dependence, dependence on hard drugs, tobacco dependence, conduct disorder, major depression, generalized anxiety disorder, fears/phobias, obsessive-compulsive disorder, mania, and positive and negative schizophrenia symptoms. Each model also included method/state factors designed to pull out age- and assessment-related variance (e.g., interviewer effects, mood effects, and age-specific vulnerabilities) that was uncorrelated with trait propensity toward psychopathology. All CFA analyses were performed in MPlus version 7.1[2] using the weighted least squares means and variance adjusted (WLSMV) algorithm.

The Correlated Factors Model reflects the observation that mental disorders form three dimensions: Internalizing (with loadings from MDE, GAD, and fears/phobias), Externalizing (with loadings from alcohol, cannabis, drugs, smoking, and conduct disorder), and Thought Disorder (with loading from OCD, mania, and schizophrenia). This model fit the data well: $\chi^2(1018, N=1000) = 1737.159$, CFI=.962, TLI=.958, RMSEA = .027, 90% confidence internal (CI) = [.024, .029]. Loadings on the three specific factors were all positive, generally high (all ps < .001) and averaged .834.

The Bi-Factor Model indicates that the ordinal symptom measures reflect both General Psychopathology (the p-factor) and three narrower styles of psychopathology (Internalizing, Externalizing, and Thought Disorders). General Psychopathology is represented by a factor that directly influences all of the diagnostic symptom factors. In addition, styles of psychopathology are represented by three factors, each of which influences a smaller subset of the symptom items. For example, depressive symptoms load jointly on the General Psychopathology factor and on the Internalizing style factor. The specific factors represent the constructs of Internalizing, Externalizing, and Thought Disorder over and above General Psychopathology. After respecification for a Heywood case (see the depiction in **eFigure B)**, the Bi-Factor Model fit the data well: $\chi^2(1012, N = 1000) = 1652.586$, CFI = .966, TLI = .963, RMSEA = .025, 90% confidence internal (CI) = [.023, .027]. Loadings on the p-factor were high (all p's<.001), and averaged .650. Similarly, the loadings for the two specific factors were also positive and averaged .651 for Externalizing and .426 for Internalizing.

The fit of these two models was very similar, and so we report results using both the General Psychopathology factor from the Bi-Factor Model as well as the Internalizing, Externalizing, and Thought Disorder factors from the Correlated-Factors Model. These factors allow us to (a) test for an overall dose-response relationship between blood lead level and General Psychopathology, as well as (b) address questions of specificity by testing for associations between blood lead level and each of General Psychopathology's three constituent psychiatric spectra. For expository purposes, we scaled Study members' scores on all factors to M=100, SD=15.

© 2019 Reuben A et al. *JAMA Psychiatry.*

Study covariates

Childhood family socioeconomic status was defined as the mean of the highest occupational status level of either parent across Study assessments from the participants' birth through age 15 years, measured using the Elley-Irving scale, which assigned occupations into one of six socioeconomic status groups (6=professional; 1=unskilled laborer; M=3.75, SD=1.14).[3]

Maternal IQ was assessed via the SRA verbal test (which is not scaled to a mean of 100) administered to the Study mothers when the Study members were 3 years old (M=39.75, SD=14.77).[4]

Family histories of mental illness were collected in 2003-2005, when the Study members were 30-33 years of age, by interviewing the Study members as well as both of their parents. As described previously,[5] family psychiatric history data were collected about each participant`s biological parents, grandparents, and siblings. Data on 7,856 family members of the Study members were used (average of 8 family members; range 3-16) to construct family histories, assessed by means of the Family History Screen (FHS)[6] and supplemented with items to broaden coverage of substance-use disorders and psychosis. A family member was considered to have a positive history of disorder if the majority of informants reported that the family member displayed at least one indicator of mental illness. Indicators included: 1) suffering from a "serious mental illness, emotional problem, or nervous breakdown," 2) ever receiving medical or psychological treatment for a mental health issue, or 3) suffering from marked functional impairment due to a mental health issue. The family history of mental illness for each Study member was calculated as the proportion of members in the family with a positive history of disorder, taking into account genetic relatedness (M=0.25, SD=0.21).

© 2019 Reuben A et al. *JAMA Psychiatry.*

eReferences

1. Caspi A, Houts RM, Belsky DW, et al. The p factor: one general psychopathology factor in the structure of psychiatric disorders? *Clin Psychol Sci*. 2014;2(2):119-137. doi:10.1177/2167702613497473

2. Muthen LK, Muthen BO. *MPlus User's Guide*. 7th ed. Los Angeles, CA: Muthen & Muthen; 1998.

3. Elley W, Irving J. Revised socio-economic index for New Zealand. *N Z J Educ Stud*. 1976;11:25-36.

4. Thurstone L, Thurstone T. *The SRA Verbal & Non Verbal Form*. Chicago, IL: Science Research Associates; 1973.

5. Milne BJ, Caspi A, Harrington H, Poulton R, Rutter M, Moffitt TE. Predictive value of family history on severity of illness. *Arch Gen Psychiatry*. 2009;66(7):738-747. doi:10.1001/archgenpsychiatry.2009.55

6. Weissman MM, Wickramaratne P, Adams P, Wolk S, Verdeli H, Olfson M. Brief screening for family psychiatric history: The family history screen. *Arch Gen Psychiatry*. 2000;57(7):675-682.

© 2019 Reuben A et al. *JAMA Psychiatry.*

eFigure. Two Alternative Correlational Structures of Adult Psychopathology

Model A: Correlated-Factors Model

Model B: Bi-Factor Model

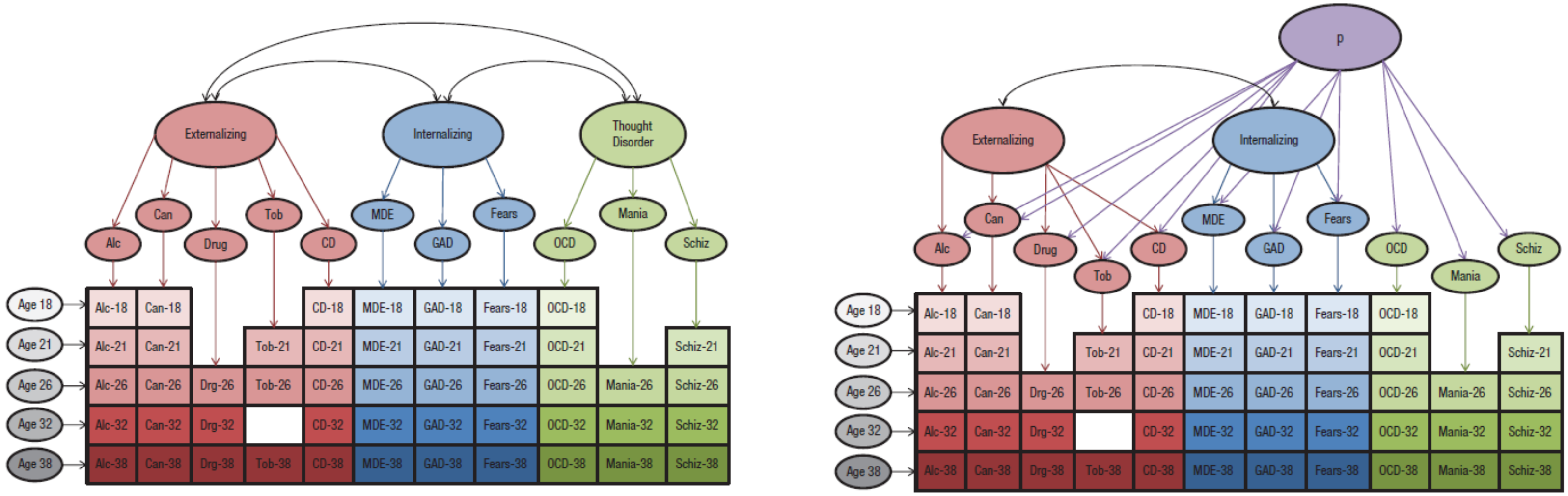

Notes. The structure of psychopathology. Colored ovals represent latent (unobserved) continuous symptom trait factors; colored boxes represent observed symptom counts for each disorder at each assessment age. The following 11 disorder/symptoms were assessed: alcohol dependence, cannabis dependence, dependence on hard drugs, tobacco dependence, conduct disorder, major depression, generalized anxiety disorder, fears/phobias, obsessive-compulsive disorder, mania, and positive and negative schizophrenia symptoms. Disorder/symptoms were assessed at ages 18, 21, 26, 32, and 38 years (not all disorders were assessed at every age, but each disorder was measured at least three times; missing assessments are depicted by white space). Gray ovals represent method/state factors designed to pull out age- and assessment-related variance (e.g., interviewer effects, mood effects, and age-specific vulnerabilities) that was uncorrelated with trait propensity toward psychopathology. Alc = alcohol; Can = cannabis; Drg = hard drugs; Tob = tobacco; CD = conduct disorder; MDE = major depression; GAD = generalized anxiety disorder; Fears = fears/phobias; OCD = obsessive-compulsive disorder; Schiz = schizophrenia. Initial tests of the Bi-Factor Model revealed that it had a Heywood case, which occurs when an estimated variance is negative for one of the lower-order disorder/symptom factors (in this case, OCD). This suggested that the original model may not be valid. Inspection of the results revealed that the Thought Disorder factor was subsumed in General Psychopathology ("p"). The model was respecified accordingly. The above figure (B) presents the respecified Bi-Factor Model. Fit statistics reported above refer to the respecified Bi-Factor Model

© 2019 Reuben A et al. *JAMA Psychiatry.*

© 2019 Reuben A et al. *JAMA Psychiatry.*
